# Supplementary material for: Efficient identification of de novo mutations in family trios: a consensus-based informatic approach
Source: Life Sci Alliance. 2025 Mar 28;8(6):e202403039. doi: 10.26508/lsa.202403039 (PMC11953573; doi:10.26508/lsa.202403039)
Supplement: Supplementary file 2 [file LSA-2024-03039_Supplemental_Data_1.docx]

SUPPLEMENTARY MATERIAL

Graph Pangenome Reference

Standard methods to recover the underlying genome from high throughput short read sequencing samples rely on a single haplotype reference genome assembly to map the sequencing reads to the corresponding generating regions, and then to identify the most likely haplotypes implied by the mapped reads. This process works well for most conserved regions in the genome but fails in regions where the haplotypes in the individual genome are dissimilar to the single haplotypes comprising reference assembly due to evolutionary or somatic divergence. This reference bias, more pronounced in non-European individuals, impairs the identification of relevant genetic mutations for appropriate clinical decision making.

Pangenome references, consisting of multiple haplotypes identified in an appropriately representative cohort of individuals, have been proposed to drastically reduce reference bias inherent in all analyses methods that use reference haplotypes. We have previously described our implementation of pangenome reference-based sequencing data analysis workflow (Rakocevic et al, 2019). We represent the pangenome as a directed graph structure with the chromosomal reference haplotype as the main path in the graph, and the alternate sequences corresponding to genomic variation represented as edges spanning variable genomic regions, as shown in Fig S2. Our representation preserves the genomic loci of genetic variants, ensuring compatibility with existing downstream workflows as well as ease of comparative benchmarking of results with standard methods. The pangenome reference was constructed by augmenting each GRCh38 chromosomal haplotype with associated alt contigs, and with high confidence variants selected from the results reported by 1000 Genomes Phase 3 study (Rakocevic et al, 2019), Simons Genome Diversity Project (Katsnelson, 2010), and other variant datasets (Mills et al, 2006; Mallick et al, 2016).

GRAF *de novo* Variant Detection Pipeline

The steps for filtering *de novo* variants for the GRAF pipeline are as follows:

1. Merge family VCFs into a multi-sample family VCF file. We used Bcftools Merge (Danecek et al, 2021) for this task (Merge Variants)

2. Identify the de novo variants within the family VCF file:

2.1. For each variant in the family VCF file, compare the child genotype with each parent’s genotype respectively. We used RTG VCFeval (Zook et al, 2016 *Preprint*) tool for this task (Compare Variants)

2.2. If the child genotype differs from either parent’s genotype, inspect each genotype triple comprising the variant call for Mendelian inconsistency to get a list of putative de novo variant loci (Collect Mendelian Violation (MV) Loci)

2.3. Use the candidate de novo variant loci to filter family VCF (Filter by Region)

2.4. Collect Mendelian violations from the family VCF filtered with candidate de novo loci. We use RTG Mendelian tool for this task (Collect Mendelian Violations (MVs))

2.5. Further reduce this list by eliminating all genotype triples where any of 6 alleles comprising the 3 sample genotypes does not have an adequate proportion of mapped reads containing evidence for the allele (Filter by Allelic Balance (AB) and Mapped Allelic Balance (MAB))

Fig S3 shows the steps for filtering *de novo* variants for GRAF pipeline.
